# *Listeria monocytogenes* bacteremia in an AIDS patient during pregnancy: A case report

**DOI:** 10.1097/MD.0000000000046352

**Published:** 2025-12-19

**Authors:** Xia Zhang, Xiang Hu, Ling Zeng, Mei Hong, Li Xiang, Yu-Man Zhang

**Affiliations:** a Department of Clinical Laboratory, Zhejiang Provincial People’s Hospital Bijie Hospital, Bijie, China.

**Keywords:** AIDS, bacteremia, *Listeria monocytogenes*, pregnancy

## Abstract

**Rationale:**

Pregnancy complicated by acquired immunodeficiency syndrome can result in a dual state of immune suppression, significantly increasing susceptibility to intracellular pathogens. *Listeria monocytogenes (L. monocytogenes*), a placenta-tropic lethal pathogen, is extremely rare in pregnant women infected with the human immunodeficiency virus (HIV). However, when it does occur, it often leads to maternal sepsis, premature birth, or fetal death.

**Patient concerns:**

The patient was a 36^+^²-week pregnant woman who presented with a 4-day history of persistent fever and was known to be HIV-positive. Laboratory tests indicated an elevated neutrophil percentage and an increased C-reactive protein level. Imaging studies revealed bilateral renal hydronephrosis. Blood culture confirmed a *L. monocytogenes* infection, identified with 99% confidence by Vitek2.

**Diagnosis:**

When pregnant women who are HIV-positive experience unexplained fever, there should be a heightened alert for potential opportunistic infections, such as *Listeria*. It is recommended that blood cultures and molecular testing be conducted within 24 hours.

**Interventions:**

Due to the patient’s allergy to *β*-lactam antibiotics, a breakthrough treatment regimen consisting of linezolid combined with imipenem and cilastatin was selected.

**Outcomes:**

After 3 days of treatment, the patient’s temperature returned to normal, and both the mother and the infant were discharged in good health.

**Lessons:**

HIV infection during pregnancy, when complicated by *L. monocytogenes* bacteremia, is extremely rare; however, it carries a very high mortality rate. This case study represents one of the few documented instances of HIV infection complicated by *L. monocytogenes* bacteremia during pregnancy. It underscores the necessity for clinicians to enhance early detection and implement precise interventions for these rare co-infections to optimize maternal and infant outcomes.

## 1. Introduction

Acquired immunodeficiency syndrome (AIDS), also known as acquired immunodeficiency syndrome, is the terminal stage of human immunodeficiency virus (HIV) infection, characterized by the progressive depletion of CD4+ T lymphocytes, leading to profound immunodeficiency.^[1,2]^ During pregnancy, the mother establishes a physiological state of immune suppression through the expansion of regulatory T cells and the expression of placental immune tolerance molecules, significantly impairing the clearance of intracellular pathogens.^[3]^The synergistic effect of dual immune suppression from HIV infection and pregnancy significantly increases pregnant women’s susceptibility to placentatropic pathogens, such as *Listeria monocytogenes (L. monocytogenes*). This bacterium invades the placenta via endocytosis and possesses intracellular survival capabilities, becoming a high-risk, life-threatening pathogen.^[4,5]^ According to the World Health Organization, the incidence of listeriosis in the general pregnant population was approximately 0.11‰ in 2023. However, the infection risk in HIV-infected pregnant women increased by 12-fold, with a sepsis mortality rate as high as 35% in those with CD4+ count < 200 cells/μL^.[6,7]^. As of 2024, global reports of HIV-infected pregnant women with concurrent *L. monocytogenes* bacteremia remain extremely rare. This report details a case of late-stage pregnancy in a woman with HIV, complicated by *L. monocytogenes* bacteremia, which was accompanied by concurrent hydronephrosis, a 72-hour diagnostic delay, and a distinctive treatment approach. The objective is to offer insights for clinical identification and intervention in comparable rare conditions.

## 2. Clinical data

### 2.1. Case profile

The patient is a female who was diagnosed with HIV infection in 2019. She presented at 36 weeks and 2 days gestation. On April 13, 2024, the patient developed a fever of unknown origin accompanied by malaise, her body temperature reaching 37.80℃. Physical cooling measures were applied to normalize her body temperature. On April 14, 2024, she had another bout of fever, which was managed with physical cooling until her temperature stabilized. On April 16, 2024, she continued to have recurrent fevers, with her temperature peaking at 38.60℃. Due to the persistent fever, she was admitted to the hospital for further evaluation and treatment. During her hospitalization, the expression of inflammatory factors was as shown in Table 1.

**Table 1:** Serum inflammatory factor levels in patients.

Time	CRP (mg/L)	WBC (×10^9^/L)	NEU (×10^9^/L)	N/L (%)
20240417	41.09	7.75	6.21	6.68
20240420	106.38	8.61	7.09	7.38
20240427	15.06	13.94	10.61	4.30
20240428	54.41	8.12	6.24	5.51
20240430	28.10	6.27	4.53	3.62
20240504	20.50	3.56	1.43	0.79
20240507	5.90	4.46	2.19	1.07

CRP = C-reactive protein, N/L = neutrophil/lymphocyte, NEU = neutrophil, WBC = white blood cell.

### 2.2. Laboratory tests

The Centers for Disease Control and Prevention have confirmed a positive HIV antibody test result. The blood test results indicate the following: Potassium is 3.15 mmol/L (normal range: 3.50–4.90 mmol/L); total protein is 67.10 g/L (normal range: 61.00–79.00 g/L); albumin is 33.60 g/L (normal range: 42.00–56.00 g/L); total bile acids are 84.35 μmol/L (normal range: 0–6.71 μmol/L); direct bilirubin is 9.90 μmol/L (normal range: 0–4.00 μmol/L); *γ*-glutamyl transferase is 74.00 U/L (normal range: 6.00–26.00 U/L); alanine aminotransferase is 114.00 U/L (normal range: 6.00–29.00 U/L); aspartate aminotransferase is 357.00 U/L (normal range: 10.00–31.00 U/L); glycocholic acid is 45.06 mg/L (normal range: 0–2.70 mg/L); and creatinine was 110.00 µmol/L (normal range: 41.00–73.00 μmol/L).The urinalysis results indicated the presence of urine white blood cells at 2+ (normal range: negative), urine protein at 2+ (normal range: negative), and occult blood at 1+ (normal range: negative). The urine culture results showed no bacterial growth.

**Figure 1.**
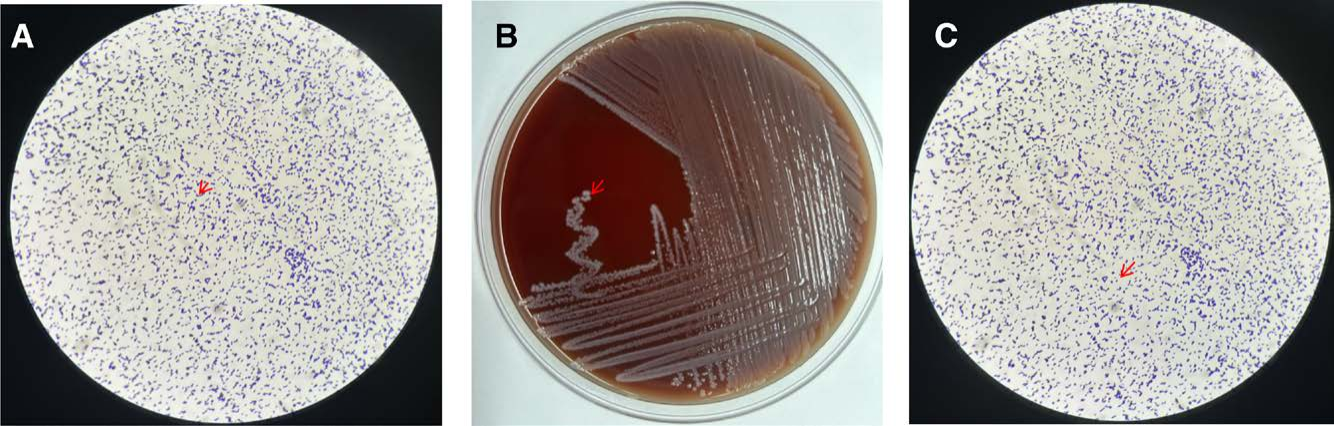
Bacterial culture results. (A) Peripheral blood culture for 1 day smear Gram staining (×1000); (B) Peripheral blood culture for 1 day and then transferred to culture at 35℃ for 48 hours; (C) Peripheral blood culture for 1 day transferred to culture at 35℃ for 48 hours smear Gram staining (×1000).

The blood test results revealed: neutrophil percentage of 80.10% (normal range: 40.00–75.00%), lymphocyte percentage of 12.00% (normal range: 20.00–50.00%), C-reactive protein level of 41.09 mg/L (normal range: 0–6.00 mg/L), and D-dimer level of 5.26 μg/mL (normal range: 0–1.00 μg/mL). The blood culture bottle signaled positive after one day of incubation. Direct smear microscopy revealed Gram-positive short rods arranged in pairs (Fig. 1A). After 24 hours of plate culture, colonies appeared grayish-white with a diameter ranging from 1.00 to 2.00 mm and produced a narrow *β*-hemolytic ring (Fig. 1B). Direct smear microscopy again showed Gram-positive short rods arranged in pairs (Fig. 1C). The strain was identified as *L. monocytogenes* with a 99% identification rate using the Vitek 2 fully automated microbial analysis system. Antimicrobial susceptibility testing was conducted in accordance with CLSI M45 guidelines, and the strain was found to be susceptible to linezolid (refer to Tables 2 and 3).

**Table 2:** Antimicrobial susceptibility results for *Listeria monocytogenes* (K-B method).

Antimicrobial agents	Results (antimicrobial zone diameter in mm)	Interpretation of results	Drug content in paper strips	Inflection point	Standard
Penicillin	26.00	S	10U	S ≥ 13.00; *R* < 13.00	EUCAST
Ampicillin	30.00	S	10	S ≥ 16.00; *R* < 16.00	EUCAST
Erythromycin	29.00	S	15	S ≥ 25.00; *R* < 25.00	EUCAST
Meropenem	32.00	S	10	S ≥ 26.00; *R* < 26.00	EUCAST

**Table 3:** Antimicrobial susceptibility results for *Listeria monocytogenes* (MIC method).

Antimicrobial agents	Results	Interpretation of results	Inflection point	Standard
Penicillin	0.25	S	S ≤ 2.00	CLSI
Linezolid	0.50	S	S ≤ 2.00	CLSI
Meropenem	0.125	S	S ≤ 0.25	CLSI
Ampicillin	0.25	S	S ≤ 2.00	CLSI

CLSI = Clinical and Laboratory Standards Institute, MIC = minimum inhibitory concentration.

### 2.3. Imaging detection

**Figure 2.**
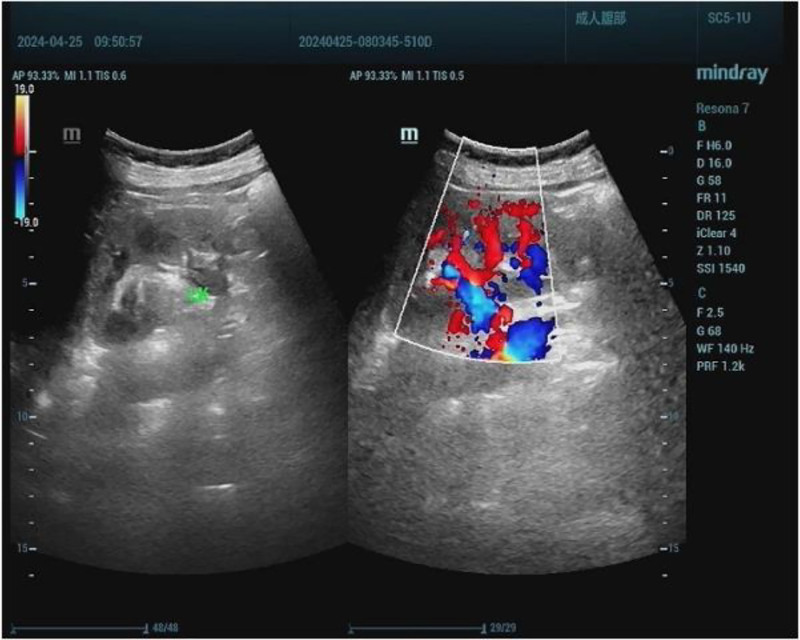
Ultrasound of the patient’s urinary system.

The ultrasound examination of the urinary tract revealed mild hydronephrosis in the right kidney and the upper part of the right ureter, as well as in the left kidney (Fig. 2).

### 2.4. Treatment and regression

The patient was diagnosed with HIV in 2019 and has been receiving antiviral therapy through oral medications: “Kaletra” (taken twice daily, 2 tablets each time), “Lamivudine tablets” (taken once daily, one tablet each time), and “Tenofovir disoproxil fumarate tablets” (taken once daily, one tablet each time). The results of a urinary system ultrasound examination revealed mild hydronephrosis in the right kidney and the upper segment of the right ureter, with similar conditions in the left kidney. Consequently, a bilateral ureteral stent placement procedure was performed under ureteroscopy to treat the hydronephrosis. Postoperatively, the patient underwent antimicrobial therapy with piperacillin-tazobactam sodium and continued taking oral antiviral medications. Electrolyte, liver function, and renal function tests indicated that the patient had severe intrahepatic cholestasis of pregnancy accompanied by hypokalemia. In response to this condition, fluid replacement, potassium supplementation, and oral ursodeoxycholic acid tablets were administered for bile acid reduction therapy. The decision was made to deliver the baby. A live male infant was successfully delivered via. However, the mother continued to experience recurrent fever postpartum, and *L. monocytogenes* was identified in a peripheral blood culture. According to Infectious Diseases Society of America guidelines, the first-line treatment for Listeria bloodstream infection is ampicillin ± gentamicin. However, due to the patient’s allergy to *β*-lactam antibiotics, an alternative regimen was initiated: intravenous linezolid (600 mg every 12 hours) for its anti-listerial activity and placental penetration, combined with imipenem/cilastatin (500 mg every 6 hours) to provide broad Gram-negative coverage and piperacillin/tazobactam sodium was replaced with imipenem/cilastatin. After 3 days of anti-infective treatment, the patient had no chills, fever, or symptoms such as frequent urination, urgency, or incomplete voiding. Treatment with linezolid tablets and imipenem/cilastatin was continued. The mother’s condition remained stable, and she reported no special discomfort. Although liver function results were still abnormal, continued hospitalization was recommended; however, the mother and her family declined further hospitalization and requested discharge. One week after discharge, a telephone follow-up revealed that neither the mother nor the infant had experienced a recurrence of fever. The newborn’s blood culture and cerebrospinal fluid polymerase chain reaction tests were negative for *Listeria*.

## 3. Discussion

AIDS is a systemic disease caused by infection with HIV.^[8,9]^ HIV primarily attacks T lymphocytes in the human immune system, resulting in a reduction in their numbers.^[10]^ This, in turn, leads to immune deficiency and an increased susceptibility to pathogens.^[11]^ Individuals infected with HIV experience a latency period of several years before progressing to AIDS.^[12]^ During this period, the infected individual’s resistance diminishes and their immunity weakens, rendering them susceptible to rare infections such as herpes zoster, fungal infections, and Mycobacterium tuberculosis.^[13]^ In summary, the cause of death in AIDS patients is not due to a single factor but rather to multiple infections resulting from a decline in immunity.^[12]^ Despite the immense efforts of medical researchers worldwide, no cure for AIDS has been developed, nor is there an effective vaccine available.^[13]^

*L. monocytogenes* belongs to the Listeriaceae family and the Listeria genus; it is a zoonotic pathogen that is prevalent in nature.^[14]^ It primarily spreads through intestinal infection, and contracting this bacterium can result in urinary tract inflammation, sepsis, and gastrointestinal inflammation.^[15]^ Symptoms often include recurrent fever, headache, muscle pain, and joint pain, which are similar to those of upper respiratory tract infections.^[16]^ The World Health Organization reports that infections caused by *L. monocytogenes* during pregnancy account for 43% of all cases, with 14% occurring in the late stages of pregnancy.^[16]^ Infections with *L. monocytogenes* during pregnancy often result in fetal death, neonatal sepsis, and neonatal meningitis.^[14]^ Individuals infected with HIV are at least 300 times more susceptible to *L. monocytogenes* infection than those with a normal immune system.^[15,16]^ Given its potentially severe consequences, healthcare providers must be knowledgeable about the impact of *Listeria* infection on HIV-positive pregnant women. Currently, case reports of bloodstream infections with *L. monocytogenes* during pregnancy in HIV-positive individuals are relatively rare.

This case report details the case of a pregnant woman with HIV (at 36^+2^ weeks of gestation) who developed *L. monocytogenes* bacteremia during childbirth. Her dual immunocompromised state significantly increased her risk of infection compared to the general pregnant population. The unique high-risk profile of this case, coupled with hydronephrosis and a 72-hour delay in diagnosis, underscores the lethality of Listeria as a placenta-tropic pathogen. To address the challenge of *β*-lactam allergy, an innovative treatment regimen that combined linezolid with imipenem/cilastatin was implemented. Leveraging linezolid’s intracellular bactericidal activity and its ability to penetrate the placenta, the maternal and infant conditions stabilized and improved within 3 days. Additionally, negative urine culture results may be influenced by the following factors: a low rate of *Listeria* colonization in the urinary tract; antimicrobial drugs administered before sample collection; and noninfectious inflammation or infection with fastidious or atypical pathogens. In summary, this case highlights the necessity of initiating the “sepsis alert pathway” for HIV-infected pregnant women presenting with fever, which includes prompt blood culture and molecular diagnosis.

## Author contributions

**Investigation:** Xiang Hu.

**Methodology:** Ling Zeng.

**Resources:** Mei Hong, Li Xiang.

**Writing – original draft:** Yu-Man Zhang.

**Writing – review & editing:** Xia Zhang.
